# Corollaries from a Treatise on the Cerebellum and Different Parts of the Brain in Epileptics

**Published:** 1815-03

**Authors:** 


*For the Medical and Physical Journal.*

*Corollaries from a Treatise on the Cerebellum and different Parts of the Brain in Epileptics;* by Professor WENZEL and his BROTHER.

IF, in closing this collection of observations, I allow myself to present some general hints, I desire they may be considered only as questions. I do not pretend to give them as incontrovertible principles in the investigation of epilepsy.

The observations which precede, perhaps, will give to some of my remarks the rare merit of entire certainty. Some of them, without doubt, will induce us to restrict ourselves a little more in the employment of remedies, and to try a more simple, and, of course, a more perfect, practice; the others will be, perhaps, of some utility in silencing the complaints which arise in consequence of the insufficiency of anatomy to elucidate the causes of the disease.

I have made these remarks because they appear to me to be true, without pretending to offer them as rules of practice. I shall receive with gratitude objections and ingenious criticisms brought against my conjectures.

I shall now consider successively all the parts of the cranium of which the author has thought it important to examine the lesions, and shall omit all those which are not directly in point.

1. The body of the base of the cranium appears in a physiological view, and more particularly in a pathological one, to merit careful research, with a view to know the state of the cerebellum.

2. There are, in this part of the bone, certain changes which ought to be considered as pathological signs, and which appear to have an essential influence on the cerebellum.

3. These changes consist either in malconformation of insulated parts, or in defect of parts in connection with them. Or the whole of the base of the cranium is affected by those malconformations.

malconformations. All these different changes may be of great importance with regard to the cerebellum.

4. It is, perhaps, in this deformity of the base of the cranium, which presses on the cerebellum, that the seat and the cause of epilepsy are found.

5. The effusion of lymph between the cranium and the dura mater, and between the folds of this membrane, occurs only in by far the smaller number of cases in which the cerebellum presents these morbid appearances.

6. All the changes in the convolutions of the brain, even those which have been most uniformly remarked, do not appear under any view to be necessary causes of epilepsy.

7. The softness or excessive hardness of the brain, the changes in the colour of the substances which compose it, cannot any longer be considered as causes of the disease.

8. We can say as much of the collection of water in the lateral ventricles, and if it be found in the bodies of epileptics, we must examine with care whether it is not rather the consequence of severe aud reiterated attacks, especially in young subjects.

9. The changes which we see in the *corpora striata*, the *thalami nervorum opticorum*, and the *tubercula quadragemina* are alike accidental, and foreign to the causes of idiopathic epilepsy.

10. The brain, at least in the greatest proportion of cases, does not exhibit any change which can be considered as an universal cause of idiopathic epilepsy.

11. The morbid state of any part of the brain appears to be rather the consequence than the cause of the general or partial convulsions. On the contrary, idiopathic epilepsy seems to have as a constant principle a morbid affection of one and always the same part.

12. The disproportion between the pineal gland and the cerebellum, and which manifests itself principally in the organic lesions of these two parts, is a point well established, according to the observations of the author.

13. It is uncertain if the morbid lesions which are discovered in the pineal gland of epileptics, are the cause or the effect of the diseases of the cerebellum: the last hypothesis is the most probable.

14. The circumstance that the pineal gland and the cerebellum are sometimes affected together, sometimes separately, depends, perhaps, more or less on the strength and energy of the cause which acts at the same time on these organs.

15. The cerebellum has been found affected in all epileptics.

16. There has almost always been discovered an effusion of lymph, more or less hard, at the point where the two lobes of the cerebellum join.

17. When this effusion, and the change which results from it, have not taken place, there is found a lesion of the superior surface of this part.

18. The cerebellum has been diseased, when all the other parts of the brain, the pineal gland excepted, has not discovered any sign of disease.

19. The most trifling changes of this part, not remarked until now, appear to have the most serious effect upon the animal economy.

20. The cerebellum appears to be, in the human body, a part more interesting than is commonly imagined.

21. It is probable, and I may say certain, that it is an engorgement of blood produced by an irritation, or an inflammatory congestion, which occasions *that collection of lymph in the brain* which we have mentioned.

22. According to the observations we have made on other parts of the bodies of animals where similar effusions are discovered, it is certain that this fluid, when it is not absorbed, undergoes by degrees all those changes which we have always remarked in similar effusions. These offer the same results that have been found in the cerebellum of epileptics.

23. Will it be granted, upon the whole, that these morbid appearances observed in the interior of the cerebellum are the cause of epilepsy? or rather only a particular class of them?

24. Will it be admitted that the epileptic attacks are the lightest, most rare, and most curable, in those cases where the morbid affections of the cerebellum are in a less degree, as in the case where there has been no effusion of lymph, or where it has been taken up by the absorbent vessels?

25. If we consider a state near to inflammation, or a real inflammation of the cerebellum and of the neighbouring parts, and the consequences of this inflammation as a cause of idiopathic epilepsy, of what efficacy are verdigrise, galls, ether, ivy, anise, antimony, assafœtida, arsenic, orange leaves, matlincon, oil of box tree, hazel tree, camphor, copper, castor, cinnabar, digitalis purpurea, glands of oaks, aromatic plasters to the head, henbane, bullock's gall, benzoin, iron, cinnamon, Venice soap, mercury, dragon's wort, tin, white vitriol, musk, figs, opium, phosphorus, hellebore, spiritus cornu cervi, gratiola, sulphur, hyssop, wood lice, flowers of zinc, rhubarb, realgar, St. Ignatius' bean, sweet marjorum, yellow amber, mutton cutlets, common salt, oil of anise, wine of rue, castration, and the butter of antimony? I ask, of what benefit were so many other ridiculous remedies which which have been recommended, such as the hoofs and horns of the elk, amulets, the flesh of the wolf, the gall of the tortoise, pulverized human teeth, a piece of silver money swallowed, the bones of the head of a horse, dog's gall, the brain of a quail rubbed on the face, the brain of a man and of a camel, a piece of the skin of a wolf, the liver of an otter, the blood of a wolf, the gall of a bear, the horn and hoof of an ass, the ashes of the mole and rabbit, the heart of the hare or the elephant, the liver of the frog, powdered placenta, the testicles of the fox, and of the ram, powdered mice, human blood, and particularly of a martyr, human excrement, the testicles of an ass, the fat of a hare, swine's snout, &c. &c. &c.?

26. Is not the inconsiderate use of these remedies, some of which may appear reasonable, but others extravagant, equally dangerous.

27. Do not incipient epilepsies demand, above all other remedies, those which have been recommended by the most respectable physicians, and which are termed antiphlogistic, particularly local and general blood-letting?

28. Are not irritating remedies dangerous, at least when the disease begins to discover itself? Do not terrible inconveniences result from them?

29. What can be expected from irritating applications to the occiput, or to the surrounding parts?

30. What can be expected in this disease, when inveterate and obstinate, from cauterising the head or neighbouring parts, which De Haen recommended, and Pouteau has again brought into vogue, and which Mandan employed with success? There is no need of repeating, that we do not treat in this place of cauterising the bones of the cranium which De Haen practised, and at first Pouteau himself, but of the integuments of the head only.

31. Should not the treatment of epilepsy, besides an examination the most exact of the nature of all the attacks since their first appearance, be preceded by serious considerations of the age, natural constitution, and the character of all the preceding diseases, of the subject?

32. Are there no epilepsies susceptible of cure but those which have for their origin the morbid affections of other parts of the body, whilst those which have their cause in the lesion of the cerebellum are, perhaps, incurable?

33. Does not constant and well-authenticated experience teach us at what period of the disease the means of our art may be best employed; and at what period the curative means may be more injurious than useful, and at the same time hazardous to the life of the patient?

34. Does not an ill-judged and wrong treatment of epilepsy in many cases render the disease incurable?

35. Will it not be advantageous to reject all those remedies which superficial reflection and uncertain experience suggest? At what period will it be best for the patient to do nothing, from the fear of hazarding too much?

36. It is a dangerous habit, which a great many presumptuous physicians have adopted, of calling those nervous diseases which they are unable to cure. The subject of epilepsy should not be left in so much uncertainty, for it well merits the trouble of being examined anew, with serious attention.

37. It will be a happy approximation towards improvement, if we cease to content ourselves with the first appearances which discover themselves after death, in dead bodies, if we constantly oblige ourselves to rely on authenticated facts, and substitute sound reasoning for the accumulated experience of quacks.

38. In what class shall epilepsy be arranged, in consequence of researches pursued in this manner?

Those alone, in my opinion, will resolve this problem, who, struck by the observations offered in this work, convinced of their truth, and having made them their own by their experience, instead of fabricating a vain system, shall take pleasure in interrogating nature, and interrogating her frequently. They will be those who will not content themselves with half observations, who will not oblige themselves to prescribe a remedy, until they have carefully examined the nature of the disease; who, in following the progress of disease, shall take for their model the conduct of great physicians; who shall do that which Wichmann has ordered in his treatise on Diagnostics, and that which Walstein prescribed before him, for the examination into the nature of the diseases of animals. These are examples well worthy of imitation.

If the horror of the disease of which we have spoken be not sufficient to engage physicians to make serious and repeated observations upon the nature and history of epilepsy, they need only cast a glance on the uncertainty and contrariety of the curative means that are opposed to it.

I do not here speak to those physicians who have for ever theories in their mouths, and who yield themselves to the most blameable empiricism, who make their reputation consist in a continual change of treatment, a change which they attribute to the improvement of the art, notwithstanding it reflects but little honour on their science or their skill. I

address address myself to those who endeavour to find relations between practical treatment and medical science; who attach themselves to the most certain data; who do not confound a vain scientific jargon with the plain language of truth; who despise the pedantic ostentation of technical terms, because there are better opportunities to employ them; who recollect that physicians as well as surgeons ought to be very sparing of their prescriptions as well as of the use of their instruments, because medicines and surgical operations ought only to be the result of an enlightened experience; who recollect and avow the imbecility of art, when not sufficiently directed by experience; who do not boast of the extent of their knowledge, when every thing announces how limited it is.

Such men will be able to throw light on that part of the science which is yet covered with darkness. The wise will admire them. The skilful physician proceeds with a steady step in the road of truth, and becomes really useful, while the half learned excites pity by his ignorance, of which, in every instance, he affords new proof.

The path which conducts to truth is not so obscure as we may believe; it is only difficult, and therefore it is that all the world cannot find it.

---

*For the Medical and Physical Journal.*

